# Selection of reference genes for qPCR in hairy root cultures of peanut

**DOI:** 10.1186/1756-0500-4-392

**Published:** 2011-10-10

**Authors:** Jose Condori, Cesar Nopo-Olazabal, Giuliana Medrano, Fabricio Medina-Bolivar

**Affiliations:** 1 Arkansas Biosciences Institute, Arkansas State University, P.O. Box 639, State University, AR 72467, USA; 2 Department of Biological Sciences, Arkansas State University, P.O. Box 599, State University, AR 72467, USA; 3 BioStrategies LC, P.O. Box 2428, State University, AR 72467, USA

## Abstract

**Background:**

Hairy root cultures produced via *Agrobacterium rhizogenes*-mediated transformation have emerged as practical biological models to elucidate the biosynthesis of specialized metabolites. To effectively understand the expression patterns of the genes involved in the metabolic pathways of these compounds, reference genes need to be systematically validated under specific experimental conditions as established by the MIQE (Minimum Information for Publication of Quantitative Real-Time PCR Experiments) guidelines. In the present report we describe the first validation of reference genes for RT-qPCR in hairy root cultures of peanut which produce stilbenoids upon elicitor treatments.

**Results:**

A total of 21 candidate reference genes were evaluated. Nineteen genes were selected based on previous qPCR studies in plants and two were from the T-DNAs transferred from *A. rhizogenes*. Nucleotide sequences of peanut candidate genes were obtained using their homologous sequences in *Arabidopsis*. To identify the suitable primers, calibration curves were obtained for each candidate reference gene. After data analysis, 12 candidate genes meeting standard efficiency criteria were selected. The expression stability of these genes was analyzed using geNorm and NormFinder algorithms and a ranking was established based on expression stability of the genes. Candidate reference gene expression was shown to have less variation in methyl jasmonate (MeJA) treated root cultures than those treated with sodium acetate (NaOAc).

**Conclusions:**

This work constitutes the first effort to validate reference genes for RT-qPCR in hairy roots. While these genes were selected under conditions of NaOAc and MeJA treatment, we anticipate these genes to provide good targets for reference genes for hairy roots under a variety of stress conditions. The lead reference genes were a gene encoding for a TATA box binding protein (*TBP2*) and a gene encoding a ribosomal protein (*RPL8C*). A commonly used reference gene *GAPDH *showed low stability of expression suggesting that its use may lead to inaccurate gene expression profiles when used for data normalization in stress-stimulated hairy roots. Likewise the *A. rhizogenes *transgene *rolC *showed less expression stability than *GAPDH*. This study proposes that a minimum of two reference genes should be used for a normalization procedure in gene expression profiling using elicited hairy roots.

## Background

Analysis of mRNA transcript levels is used to study gene expression profiling in organisms. Different techniques are employed to achieve this goal. Among them northern blot and *in situ *hybridization [1] and RNAse protection assays [2] have been the most used. Even though these techniques have some disadvantages, they are all still in some use. Polymerase chain reaction (PCR) technology emerged in the 1980s and from that period different techniques were developed using this powerful and highly sensitive platform. Several of these PCR-based techniques were developed to address gene expression analysis. One of the most widely adopted procedure involved reverse transcription PCR (RT-PCR) [3,4], where a reverse transcriptase reaction (generation of cDNA from RNA) is performed followed by a PCR and then the amplicons are visualized by gel electrophoresis [5]. The main drawback of conventional RT-PCR is the analysis of the amplicons at the end-point (plateau level) of the PCR amplification and thus gene expression analysis is only semi-quantitative. In the early 1990s, real-time PCR technology using fluorescent dyes allowed the processes of amplification and detection of a target to be monitored in real-time. The MIQE (Minimum Information for Publication of Quantitative Real-Time PCR Experiments) guidelines [6] refers to real time reverse transcription quantitative PCR as RT-qPCR, which is currently the most sensitive and widely used method for accurate determination of gene expression profiling. The advantage of this technique is that the kinetics of the reaction is measured in the early (exponential) phase of PCR, thereby providing higher sensitivity, reproducibility and a broader quantification range than previous molecular techniques [5,7,8]. Detection of amplification in qPCR is achieved when the fluorescence of a sample crosses the threshold (baseline above fluorescence background). The cycle at which the fluorescence from the sample crosses the threshold is called the quantification cycle (Cq). The concentration of the amplified gene will determine early or late Cq values for high or low expression of the gene, respectively.

Regardless of the technique used for gene expression analysis, data normalization is crucial to get accurate and reliable gene expression measurements. Normalization is used to correct variation associated with variability along the multistep experimental procedure (e.g. sample to sample variation, errors in sample quantification, RT-PCR efficiency). Different normalization strategies have been proposed [9] and, among them, the use of endogenous unregulated reference gene transcripts is the most common method [6,10,11]. Reference genes (previously known as housekeeping genes) are internal controls which are exposed to all sources of variations throughout the assay in the same way as the gene of interest. In an ideal scenario, the gene expression profile of a reference gene should not be influenced by the experimental conditions. However, many of the genes used as conventional reference genes for quantification of mRNA expression including glyceraldehyde-3-phosphate (*GAPDH*), α-tubulin (*TUBA*), β-actin (*ACTB*) and 18S ribosomal RNA (*18S rRNA*) have been shown to vary in expression levels under different experimental conditions [12-16] which leads to misrepresentation of target gene expression. Therefore, the normalization strategy using an internal control is greatly dependent on the reference gene employed and consequently the use of a reference gene without previous validation under the specific experimental conditions can lead to inaccurate data interpretation and to erroneous expression levels of target genes [6,17].

**Figure 1:**
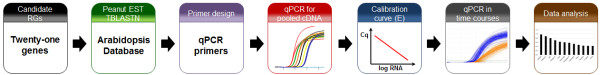
**Scheme of experimental design**. Twenty-one candidate reference genes were evaluated for qPCR in peanut hairy root. Peanut nucleotide sequences were obtained using homologous sequences from *Arabidopsis*. Then, primer design was performed using AlleleID 7 software. The set of primers for the 21 candidate reference genes were tested in a pool of samples from peanut hairy roots. PCR efficiency was calculated and used as first screening to select the genes to be analyzed in two time course experiments in peanut hairy roots. Data analysis was performed to determine the best reference gene and optimal number of reference genes to be used for normalization procedures.

To date there is not a well-defined and validated set of reference genes described for peanut (*Arachis hypogaea*) that show stable expression across a range of experimental elicited conditions. In the present study, we tested 21 candidate reference genes for qPCR. They were evaluated in a time course experiment under two abiotic stress (elicitor) conditions (NaOAc and MeJA) in peanut hairy root cultures. A schematic summary of this procedure is summarized in Figure 1. Validation of reference genes was done following the MIQE guidelines. To our knowledge, this is the first study done to validate reference genes in hairy root cultures.

## Results and discussion

In recent years, concerns about validation of reference genes and reproducibility of qPCR experiments have increased. To this end, the MIQE guidelines [6,18] recommend which information should be provided in a publication in order to make the qPCR experiments reproducible. Some key issues in the qPCR process are sample quality, PCR efficiency, number of reference genes used for normalization and validation of these reference genes. Quality, purity and quantification of RNA are important because they affect the entire RT-qPCR process [19-22]. PCR efficiency is calculated using different RNA concentrations and fluorescence values generated at specific RNA concentration. The fluorescence is due to a fluorescent dye (SYBR Green), which is in complex with the double strand cDNA. In an ideal scenario the efficiency should be 100%, it means that the amount of product is doubled with each PCR cycle. PCR efficiency determines the performance of the PCR assay which involves purity of the template and optimum PCR amplification conditions. In more detail, PCR efficiency is affected by the quality of the template (cDNA, DNA), template concentration, low expression of the gene, primer design (unspecific PCR products), cycle conditions [23-25]. Therefore, reporting detailed procedures across qPCR can guarantee that it can be reproduced by others.

In the last years a number of studies on the validation of reference genes have been done for different plant species [26-34]. Most of them used a list of reference genes from other plant species and tested them under their experimental conditions. Then software-based applications such as geNorm [35], NormFinder [36], BestKeeper [37] or qBase [38] were used to perform statistical identification of the best reference gene from a group of candidate genes in a defined set of biological samples.

Plants produce a wide range of phenolic compounds which are derived from the phenylpropanoid/acetate pathway [39]. Many of these metabolites are produced by the plant under pathogen attack and appear to function as phytoalexins [40,41]. Among this group of inducible phenolics are the stilbenes (also referred as stilbenoids), which recently have caught the attention of scientists because of their numerous health benefit properties [42-45]. Interestingly, stilbenes are found in non-related taxonomically plant species such as peanut and grapevine [46]. Previously, our laboratory showed that peanut hairy root cultures are a good model system to study the biosynthesis of stilbenoids [47,48]. To our knowledge only a single study was done with grape where reference genes were validated to study the expression profile of the stilbenoid metabolic pathway [34]. In the case of peanut, few studies have been conducted using qPCR. In two of them, ubiquitin [49] and actin [50] were employed as reference genes. However, none of these studies validated their use. Sequencing of the genome of model plant species such as *Arabidopsis *has provided a starting point to identify homologous reference genes in other species where no genome sequence is available. Also, microarray data for *Arabidopsis *has been used to identify new reference genes. This study showed that conventional reference genes are often not a good choice [13].

### Candidate reference genes

Previously we showed that abiotic stressors (also referred to as elicitors) can induce the production of stilbenoids in peanut hairy roots [47,48]. To identify reliable reference genes to be used in gene expression studies in elicited hairy root cultures, a time course experiment was designed using 2 well described elicitors NaOAc and MeJA, shown to stimulate stilbenoid production. Nine time points (0, 1, 3, 6, 12, 24, 48, 72 and 96 h) per treatment and two non-elicited control points (0 and 24 h) were considered. Due to the unavailability of plant databases for qPCR reference genes, we searched the literature for qPCR reference genes used in plants (additional file 1). Six genes were classified as conventional reference genes (*actin, tubulin, elongation factor, 18S rRNA, GAPDH *and *ubiquitin*) because they are the most frequently used genes. Based on microarray data from *Arabidopsis*, Czechowski et al. [13] proposed new candidate reference genes, which later were validated in other plant species (additional file 1). Comparison of plant and animal reference genes showed that in most cases, the same genes were used. From the summary done based on the literature review (additional file 1), 19 reference genes were selected. These genes showed different roles in the cell that include cell structure, membrane proteins, transcription, protein translation, protein folding, protein degradation (proteasomal) and metabolic pathways. This selection was done in order to reduce the chance that the genes could be co-regulated and therefore involved in similar functions in the cell (Table 1). Also the idea of using transgenes as reference genes was explored. The hairy root phenotype is due to the expression of bacterial genes that are integrated into the plant nuclear DNA. In the case of *Agrobacterium rhizogenes *strain ATCC 15834 two clusters of genes are mainly responsible for the hairy root phenotype: the *rol *[51,52] and *aux *[53] genes. From the cluster of *rol *genes, *rolC *was targeted because it was found to be expressed at higher levels than *rolA *and *rolB *genes in root tissue [54]. Knowing that the T-DNA integration into nuclear DNA starts from the right border (RB) [55,56], the *aux1 *gene was targeted as a reference gene because it is closer to RB on the T_R_-DNA and therefore it should have a better chance for the integration than *aux2*. Furthermore, the presence of these genes in the peanut hairy root line 3 used in this study was previously confirmed by PCR [48]. In total, 21 candidate reference genes were evaluated (Table 1).

**Table 1:** Reference genes selected, primer sets and amplicon characteristics for qPCR

Gene name	Peanut GenBank Accession	Arabidopsis				Primer pair 5'-3' (forward/reverse)	Length (bp)
				
		Homologous locus	Locus Description/Function	Intron/number of introns	Amino acid identity with peanut (%)		
*TUA3*	EZ722876.1	AT5G19770.1	Tubulin α-3/structure (cytoskeleton)	Yes/4	98	ATGGAATGATGCCTAGTGACA/ CTTGCCGACGGTGTAGTG	239
*ACT7*	EZ723877.1	AT5G09810.1	Actin 7/structure (cytoskeleton)	Yes/4	98	ATGTATGTAGCCATCCAAG/ ACCAGAGTCCAGAACAATA	75
*TIP41*	EZ743809.1	AT4G34270.1	TIP41-like family protein/membrane protein	Yes/7	70	TTATGATGAGGTAGTCCTATATG/ AACTCTAAGCCAGAATCG	121
*SAND*	EZ757404.1	AT2G28390.1	SAND family protein/membrane protein	Yes/13	74	TTGACGATGATACATACTTG/ TTCACTAAGGACATTGGA	116
*H3*	EZ727285.1	AT3G27360.1	Histone H3/DNA binding. Nucleosome assembly	None	100	ACTAACCTCTGTGCTATT/ TAGAATCTGAATCTGAATCTC	109
*TBP2*	EZ735662.1	AT1G55520.1	TATA binding protein 2/ TATA-box binding protein. Required for basal transcription	Yes/9	92	GAGTGAGCAACAGTCTAA/ TCTGGTTCATAACTTGAGA	183
*RPB1*	EZ751167.1	AT4G35800.1	RNA polymerase II large subunit/ DNA-directed RNA polymerase activity, DNA binding	Yes/13	91	CTATTGGAACTGGAGAAT/ AATAACTTGGAGACATCA	168
*EFα1*	EZ728203.1	AT5G60390.1	Elongation factor α1/ calmodulin binding, translation elongation	Yes/2	95	GGTGTCAAGCAGATGATT/ ACTTCCTTCACGATTTCA	92
*RPL8C*	EZ730256.1	AT4G36130.1	60S ribosomal protein L8/structural constituent of ribosome	Yes/1	92	GATAACGATACCTCTAGGA/ AACTTGACCAATCATAGC	84
*EIF4A1*	EZ730703.1	AT3G13920.1	Eukaryotic translation initiation factor 4A1/translation initiation factor	Yes/4	92	AACATCAATATCAACATCATCAT/ AAGAAGTCCTGTCCATCA	126
*CYP1*	EZ735209.1	AT4G34870.1	Peptidyl-prolyl cis-trans isomerase (cyclophilin)/ protein folding, signal transduction	None	81	GTGGCTCTGATACCTTAA/ ATATGTCTTAGTTGTCATTACC	177
*GAPDH*	EZ732115.1	AT1G13440.1	Glyceraldehyde-3-phosphate dehydrogenase/Glycolisis-Gluconeogenesis	Yes/10	92	TCTCTACTACTCACTCTTCT/ TTCTTCCGAATCCGTTAA	91
*APT1*	EZ730819.1	AT1G27450.1	Adenine phosphoribosyltransferase/Purine metabolism	Yes/6	80	TGCTAACAATTCTCATCT/ AACATAATTCCAGGCTTA	161
*UBQ11*	EZ726839.1	AT4G05050.1	Ubiquitin 11/ protein binding	Yes/1	100	GACTACAACATCCAGAAG/ GGTAAGGGTCTTAACAAA	84
*AP47*	EZ735656.1	AT5G46630.1	Clathrin adaptor complexes medium subunit/Endocytic pathway	Yes/12	91	TTTGGTTTGGAAGATTAGGA/ ATGCTGTGAACATTGGAA	146
*AT4G33380*	EZ737276.1	AT4G33380.1	Expressed sequence	Yes/6	77	CACAAACATCAGGAATGCT/ CAGACGGATGCGAATTTC	97
*PP2AA3*	EZ723724.1	AT1G13320.1	Protein phosphatase 2A subunit A3/regulatory subunit of protein phosphatase 2A (PP2A)	Yes/12	88	AAGGACAAGGTATATTCAA/ GTCATCCGATACAGATAA	146
*HEL*	EZ753755.1	AT1G58050.1	Helicase domain-containing protein/helicase activity	Yes/33	70	AAAGGTTGAAACAAACAGAGTAT/ CAAGTCCAGTCTGATGCT	106
*AT1G31300*	EZ752355.1	AT1G31300.1	Expressed sequence	Yes/8	74	TACCACGTCTACCTGCACTAT/ TGGCACTACAATAACTAGCATTTC	75
rolC*		K03313.1	Root loci C/auxin sensitivity. From pA4	None		GACCTGTGTTCTCTCTTT/ TCCTTCTTCGTCAATATCC	147
aux1*		DQ782955.1	Tryptophan 2-monooxygenase/ auxin synthesis pathway. From p15834	None		TATTTGAAAGTGGGTTTATC/ AATTCCTTCGTAACTCAG	83

*Sequences from *A. rhizogenes *strains A4 and ATCC 15834 were used to design qPCR primers.

Identification of intragenic regions in the peanut sequences was done with the aim of designing qPCR primers to flank those regions. This strategy was useful to detect DNA contaminants in RT-qPCR. In this case, primers span intron region(s) that results in little to no amplification of genomic DNA template under qPCR conditions. This concept has been also used in previous studies [30,57]. The *Arabidopsis *sequence was used as a model since gene annotations are available for this species. All the analyzed genes presented introns in their sequences with the exception of *H3 *and *CYP1*. The number of introns present in *Arabidopsis *sequence for each reference gene tested ranged from one (*RPL8C *and *UBQ11*) to 33 (*HEL*) (Table 1). On the other hand, amino acid identity between *Arabidopsis *and peanut of the 19 sequences had a mean value of 87.11% (SD = 10.21%). A 100% amino acid identity was found for *H3 *and *UBQ11*, whereas the lowest identity (70%) was present in *TIP41 *and *HEL*. In the current study, a higher amino acid identity for our selected genes was obtained when compared to a previous study in which amino acid from tomato was compared to *Arabidopsis*. In that study, amino acid identity between tomato and *Arabidopsis *ranged from 61.4 to 95% [30]. In the case of potato during biotic and abiotic stress [27], the highest similarity between potato EST and *Arabidopsis *was 83%, at the nucleotide level. In the present study, primer pairs targeted a single gene for members of gene family (*GAPDH *and *UBQ*). This approach was different to the one used for qPCR primers in grapes [29], where a primer pair was designed to target more than one gene family member.

Peanut sequences obtained for the 19 candidate reference genes (after TBLASTN) belonged to contig sequences from a Transcriptome Shotgun Assembly (TSA) for *Arachis hypogaea *(Institute for Plant Breeding at University of Georgia and submitted to the GenBank in 2010). These assemblies derived from SRA (sequence real archive) and ESTs (expressed sequence tag).

### Selection of RNA extraction method

RNA extraction methods can produce different RNA yields depending on the characteristics of the plant and/or tissue employed. In efforts to establish an optimal protocol for peanut hairy roots, two different methods of RNA extraction were evaluated: Maxwell^® ^(Promega) and TRIzol^® ^(Invitrogen). Maxwell^® ^uses guanidine thiocyanate to lyse samples, denature nucleoprotein complexes and inactivate ribonucleases. This broader and selective binding of RNA to the resin enriches the RNA for template. The Maxwell^® ^instrument uses plungers designed to capture and release coated paramagnetic particles attached to biomolecules (RNA) into wells of prefilled reagent cartridges. On the other hand, the principle of TRIzol^® ^method which also uses guanidine isothiocyanate for inactivation of RNases, relies on acidic phenol/chloroform for the partitioning of RNA into an aqueous supernatant for separation. It has been reported that isolation of RNA from woody plants/tissues with high levels of polyphenols and polysaccharides is challenging [58,59]. In this study, peanut hairy root tissue was employed as material for RNA extraction. Based on our experience, peanut hairy roots contain high levels of phenolic compounds. Therefore, β-mercaptoethanol (BME), which has been recommended for use in plants with high levels of phenols/tannins during DNA extraction, was tested in the Maxwell^® ^procedure. The manufacturer also recommends BME for mammalian tissue with high levels of nucleases. In addition, the amount of lyophilized material used for RNA extraction was evaluated in order to determine the capacity of the system. For this purpose three amounts of starting material were tested: 10, 20 and 40 mg dry weight (DW).

**Figure 2:**
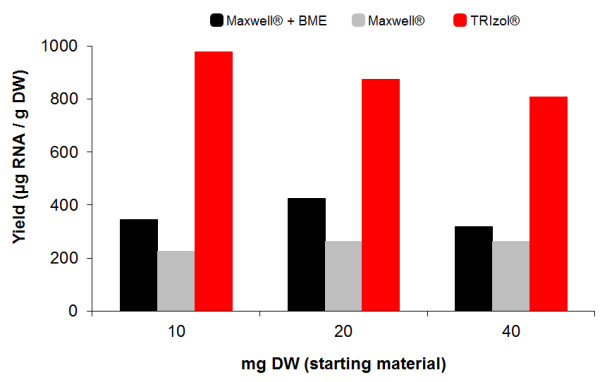
**Comparison of RNA extraction methods**. RNA yields under three different extraction methods: TRIzol^®^, Maxwell^® ^and Maxwell^® ^plus the addition of BME. Three amounts of peanut hairy root were evaluated: 10, 20 and 40 mg DW. Lyophilized tissue of 9-day old root culture was extracted under each method. RNA was quantified by Quant-iT™ RiboGreen^® ^RNA kit. Data shown represents a single replicate per method at 3 distinct amounts of starting material.

As shown in Figure 2, the highest yield of RNA was obtained with the TRIzol^® ^method (more than 2-fold when compared to Maxwell^®^), independent of the amount of starting material. In the case of TRIzol^®^, the amount of recovered RNA decreased when the amount of starting material (DW) increased, indicating that saturation of the system could be reached using 20 mg DW. Based on these results, we decided to use TRIzol^® ^as the RNA extraction method, since it provided the best RNA yield. Another advantage of TRIzol^® ^is that the time to complete the extraction procedure is faster than Maxwell^®^; although the latter uses an instrument to automate the processing of up to 16 samples, a pre-process of samples is required for RNA extraction.

### Establishing quality control parameters for RNA samples

**Figure 3:**
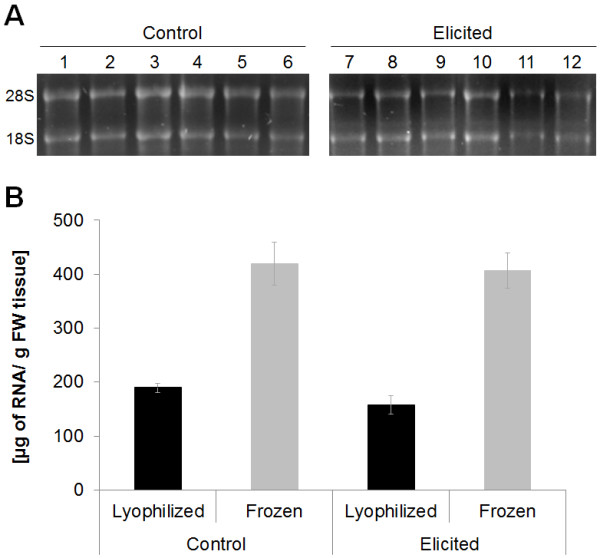
**Comparison of integrity and yields of RNA between lyophilized and frozen peanut hairy roots**. (**A**) RNA integrity was compared between lyophilized and frozen material. The two ribosomal subunits (28S and 18S) are indicators of RNA integrity on agarose gel electrophoresis. Peanut hairy roots elicited with NaOAc and non-elicited (control) were also compared. Numbers 1 to 12 represent biological replicates. (**B**) RNA yields were compared between these two conditions. For lyophilized tissue, RNA yields were expressed based on fresh weight (FW). Bars represent the average of three biological replicates and error bars represent the standard deviation. Total RNA from 9-day old root cultures was extracted with TRIzol^® ^and quantified spectrophotometrically as described in Methods section.

Assessment of all RNA samples quality is required to verify that templates for RT-qPCR are in good condition and of equivalent quality. The MIQE guidelines [6] include a section for reporting integrity and quality of RNA. In our study, lyophilized tissue was used for RNA expression analysis. Lyophilization (freeze-drying) has been reported to have an effect on RNA integrity [60]. The lyophilization process is thought to limit or reduce degradation of cellular components by inactivation of proteolytic enzymes and nucleases. To test if our lyophilization procedure [48] affected peanut hairy root RNA integrity, we compared frozen versus lyophilized tissue, as starting materials for RNA isolation. In addition, we tested if elicitation conditions (NaOAc) also affected RNA integrity. Integrity of the RNA isolated from lyophilized and frozen elicited and non-elicited hairy root tissue was compared in Figure 3A. Agarose-formaldehyde gel electrophoresis resolved the two ribosomal RNA subunits (28S and 18S), showing intact RNA. As reported by others, no effect of the lyophilization process was found in this study [61,62]. Also, we showed that there was no effect of elicitation treatment on RNA integrity. Compared to lyophilized tissue a 2-fold increase in RNA yield was obtained from frozen tissue (Figure 3B). The difference of RNA yield could be because 0.31 g (SD = 0.03) FW of lyophilized tissue was used instead of 0.1 g FW as used for frozen tissue. Therefore, the use of 0.3 g FW was beyond the capacity of the TRIzol^® ^method for RNA extraction. RNA yields were significantly (p < 0.05) different between frozen and lyophilized tissue, in control and elicited samples. On the other hand, there was not significant (p < 0.05) difference between control and elicited samples, in frozen and lyophilized tissues.

**Figure 4:**
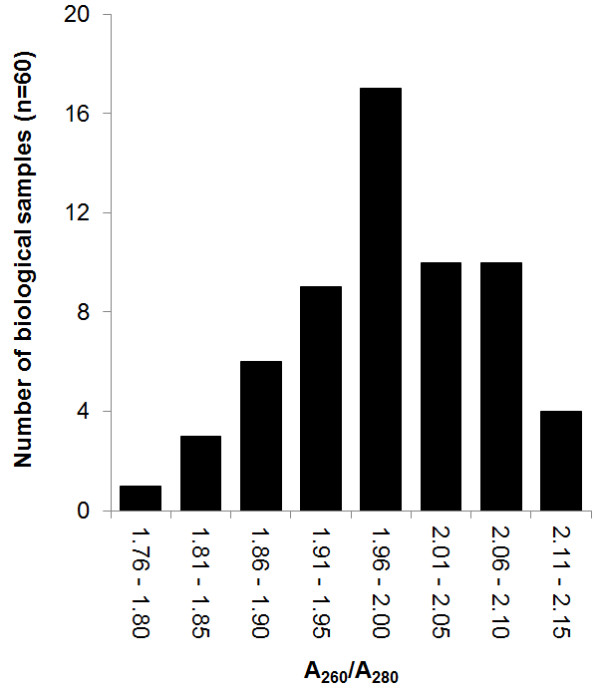
**RNA purity: A_260_/A_280_**. Purity of RNA was measured using the ratio of absorbance at 260 nm/absorbance at 280 nm. X- axis shows the intervals of A_260_/A_280 _for the 60 biological samples from the time course experiments.

It is important that RNA is free of contaminants such as proteins, DNA, cellular material, and reagents associated with RNA extraction (phenol, ethanol and salts). The impurity of RNA can affect the efficiency of RT-PCR resulting in reduced amplification. RNA purity was determined using the A_260_/A_280 _ratio. As shown in Figure 4, our samples had an A_260_/A_280 _ratio between 1.76 and 2.15. Values for the A_260_/A_280 _ratio had a mean value of 1.98 (SD = 0.06). Most of the samples were at the A_260_/A_280 _ratio of 1.96-2.00. All of the A_260_/A_280 _values fell in the range of 1.7 - 2.1 as recommended for qPCR by the manufacturer (Ambion^®^) (TechNotes 11, Applied Biosystems). The A_260_/A_230 _ratio also is used as indicator of chemical contaminants in nucleic acids. Polyphenols have been indicated as inhibitors in plant material for RT, PCR and qPCR [63]. Some phenols have absorbance near 230 nm, which decrease the expected A_260_/A_230 _value of 2.0-2.2 (NanoDrop, Technical support bulletin T009). In the present study, the mean A_260_/A_230 _value was 0.56 (SD = 0.17) (additional file 2) which is lower than the expected value (~2). These low A_260_/A_230 _values can be justified because all the RNA samples were treated with TURBO DNA-*free*™, which indicates the presence of significant absorbance at ~230 nm due to the enhancer in the TURBO DNase Buffer (TURBO DNA-*free *kit, Ambion). Inhibitors are present in all RNA samples independent of the RNA purification method used. Whether a specific compound is considered inhibitor depends of the concentration at which it disturbs the qPCR reaction. Inhibition is related to high concentration of specific compounds. In qPCR, it is important to be below the inhibition range in order to avoid their effect [64].

### Assessment of qPCR primer performance

**Figure 5:**
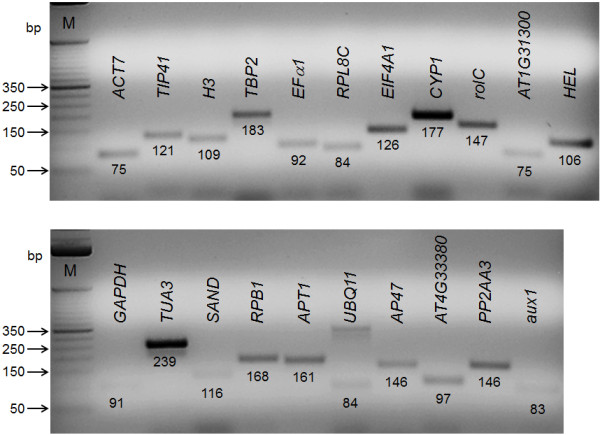
**Performance of primer amplification**. Amplicons obtained from qPCR endpoint analysis were separated by agarose gel electrophoresis. Target genes corresponding to test primer sets are indicated above the amplicon and the expected size in base pairs (bp) of each amplicon is shown.

A total of 21 genes were selected as candidates for reference genes in peanut hairy roots (Table 1). A pool of 6 samples from the time course experiment upon NaOAc treatment was used to run calibration curves with the 21 genes for qPCR using SYBR Green. After qPCR amplification, amplicons were analyzed on agarose gel to verify their product size (Figure 5). Most of the amplicons showed a single band of the expected size (Table 1). Only in the case of *UBQ11 *more than one band was observed. Consequently, melting curve identified two peaks (additional file 3), which correlated to the observations on the agarose gel. For the remaining genes, melting curve analysis was also performed and a single peak was observed (additional files 4, 5, 6, 7, 8 and 9). Figure 5 shows that intron prediction in peanut sequences for the 14 genes that contain introns was accurate, because a single expected band size by agarose gel electrophoresis was obtained. Amplification primers were tested using genomic DNA as template. Amplicons were longer than observed with a cDNA template, or no amplicon was observed for some genes (data not shown).

The melting temperature depends on base composition and length of the amplicon. In this study, average melting temperature (22 peaks, considering the second peak for *UB11*) was 80.39°C (SD = 2.579). Melting temperatures ranged from 77.50°C (SD = 0.000) for *GAPDH *to 88.03°C (SD = 0.129) for *TUA3*, indicating that amplicon sequences were different between the candidate reference genes. Melting curves with peaks lower than 78°C could indicate the presence of primer dimers in the reaction or alternatively smaller non-specific amplicon products or high AT-rich amplicons could produce lower melting temperatures. These analyses were performed using the data that was generated to determine PCR efficiencies. *UBQ11 *presented two peaks, one of them at 85.50°C (SD = 0.000), and the other at 78.63°C (SD = 0.226). For UBQ11 the presence of two peaks could indicate the presence of primer dimer or also the amplification of other mRNA sequence. In the remaining 20 genes no contaminating products (contaminating DNA or primer dimers) were present in the reaction, because no additional peak separate from the desired amplicon peak was observed. The amplicon size for *ACT7 *was 75 bp (Figure 5), which corresponded to the expected size if no genomic DNA was present (Table 1). If a contaminant genomic DNA was present in the template, an amplicon of 162 bp should be observed, but this was not the case (Figure 5). This result confirmed that no contaminant genomic DNA was present in our samples and that the treatment with DNase was effective in removing genomic DNA.

### Efficiency of reference genes

Efficiency for each of the 21 candidate reference genes was calculated. Efficiency was determined to test the performance of the PCR assays, and then used as our first criterion to screen our set of genes. In this case, only primers that fell in the range 90 - 110% of efficiency and that showed an r^2 ^≥ 0.95 were considered for further analysis. Efficiency analysis was performed using qbasePLUS software. Table 2 shows that only 11 of the 21 genes had these requirements. There was a wide range of efficiencies from 83.5% for *AT4G33380 *to 602.1% for *aux1*. Three genes (*TIP41, HEL *and *AT1G31300*) out of the seven that came from the transcript array in *Arabidopsis *[13] were considered for further analysis. The gene with the closest 100% PCR efficiency was *AT1G31300 *(99.9%). This gene was selected from the supplementary information for stressed roots from the transcript array study in *Arabidopsis*, which showed less variation under different conditions [13]. The function of *AT1G31300 *is unknown even in *Arabidopsis *and this is the first time where this gene has been used as candidate for reference gene selection in plants. From the 5 traditional genes used as reference gene (additional file 1), 2 genes (*ACT7 *and *EFα1*) were selected. From the transgenes, *rolC *was considered as reference gene but not *aux1*. Amplification of NTC was observed for 2 genes (*UBQ11 *and *aux1*). However, these genes were also excluded as candidates for reference genes, because their efficiencies were not in the range of 90 - 110%. In summary, we considered 11 genes (*ACT7, TIP41, H3, TBP2, EFα1, RPL8C, EIF4A1, CYP1, HEL, AT1G31300, rolC*) that met the expected requirements as already explained. Interestingly, when a calibration curve for *GAPDH *was done with 5 points (from 8 to 0.0128 ng) instead of 6 (from 40 to 0.0128 ng), it showed an efficiency of 95.6% and r^2 ^= 0.986. Even though the range of template concentration was below the concentration (10 ng) used in the time courses; we chose to target *GAPDH *along with the previous 11 selected genes to establish those reference genes that are not impacted by treatment over time course used in our elicitation studies.

**Table 2:** PCR efficiency of candidate reference genes

Target gene	**Efficiency**^**a**^	**SE (E)**^**b**^	**Slope**^**c**^	**r**^**2 d**^	**NTC**^**e**^	**Reference gene rating**^**f**^
*TUA3*	116.3	0.037	-2.985	0.911	N/A	Poor
*ACT7*	99.1	0.012	-3.344	0.979	N/A	Excellent
*TIP41*	93.2	0.007	-3.497	0.991	N/A	Good
*SAND*	128.4	0.040	-2.787	9.190	N/A	Poor
*H3*	99.8	0.009	-3.328	0.987	N/A	Excellent
*TBP2*	107.2	0.026	-3.161	0.919	N/A	Good
*RPB1*	414.8	1.261	-1.405	0.218	N/A	Poor
*EFα1*	102.3	0.008	-3.268	0.991	N/A	Excellent
*RPL8C*	95.2	0.008	-3.442	0.990	N/A	Excellent
*EIF4A1*	107.0	0.017	-3.166	0.970	N/A	Excellent
*CYP1*	92.4	0.006	-3.519	0.993	N/A	Excellent
*GAPDH*	124.2	0.030	-2.852	0.925	N/A	To be considered
*APT1*	155.7	0.072	-2.453	0.884	N/A	Poor
*UBQ11*	114.9	0.018	-3.010	0.967	31.08	Poor (dimer)
*AP47*	86.8	0.008	-3.686	0.986	N/A	Poor
*AT4G33380*	83.5	0.028	-3.795	0.859	N/A	Poor
*PP2AA3*	120.7	0.051	-2.908	0.797	N/A	Poor
*HEL*	101.2	0.011	-3.293	0.983	N/A	Excellent
*AT1G31300*	99.9	0.008	-3.324	0.991	N/A	Excellent
*rolC*	105.2	0.012	-3.203	0.983	N/A	Good
*aux1*	602.4	0.568	-1.181	0.771	33.79	Poor

^a^, (%) determines the performance of the PCR assay. ^b^, Standard error of the efficiency. ^c^, Slope calculated between log_10_RNA (ng/reaction) and Cq values. ^d^, Determines the degree of linear correlation between log_10_RNA and Cq values. ^e^, No template control. ^f^, Observation was done based on efficiency values, NTC and melting curves analysis. Values were calculated using qbasePLUS.

### Variability of quantification cycle (Cq) values between treatments

It is important to consider several aspects of a RT-qPCR experiment when establishing normalization parameters. Typically, variability in qPCR comes from two sources: the model system (biological variance) and the work flow (technical variability). High variability can be present in living organisms (e.g. tissue complexity, type of tissue, genetic variability, environmental impact, and species), which needs to be controlled when carrying out quantitative analyses like RT-qPCR. On the other hand, compared to biological variance the technical procedures (e.g. sampling techniques, tissue storage, RNA purification method, RT reaction, qPCR reactions, real-time instrument performance, and normalization procedure adopted) typically show less variability and are easier to control. Identification of which variable is the source of most of the variability is important. We focused on the technical procedure (technical variability), in which many of the qPCR studies have used technical replicates at the qPCR level, i.e. after the RT process where each cDNA is loaded in replicate into the plate to run real-time PCR (technical replicates). Even though there is a contribution of variability at this level, most of the variability in a RT-qPCR experiment comes from the RT step [65]. Statistically, it has been shown that qPCR replicates are not necessary because only technical variation is measured [66]. Considering these facts, we decided to use replicates at the RT level for the 12 genes in our time courses; for statistical purposes 3 RT replicates were employed.

**Figure 6:**
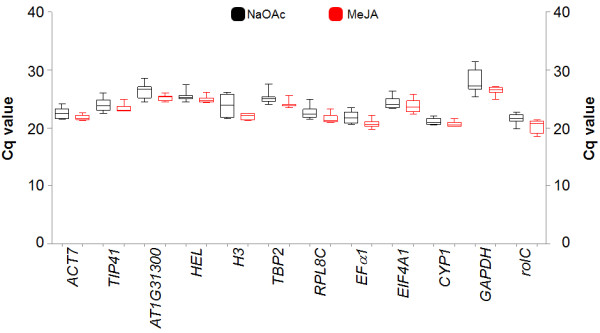
**Box-and-whisker plot showing the expression profile of reference genes under different elicitation conditions**. RT-qPCR expression values for candidate reference genes in peanut hairy root treated with NaOAc or MeJA. Expression data are displayed as cycle threshold values for each (Cq). The median quartiles and minimum and maximum Cq of the 60 samples were calculated using GraphPad Prism^® ^software.

Quantitative PCR (qPCR) was performed for the two time course experiments (9 time points per treatment) using the 12 candidate reference genes selected. Inter-plate calibrator, which corrects for variation between runs due to instrument settings, was used. Comparison of Cq values for each candidate reference genes was not significantly (p > 0.01) different between the two treatments (Figure 6). Analysis of variance of Cq values for each gene between treatments was done assuming normal distribution along the data. Variation of Cq values was higher in the NaOAc than in the MeJA treatment. This showed that gene expression under MeJA treatment is less variable than under NaOAc over the defined elicitation time used in the study. Independent of the treatment, the highest Cq value (31.42 for NaOAc and 27.16 for MeJA) was observed for *GAPDH*; while, the lowest Cq value (19.89 for NaOAc and 18.51 for MeJA) was for *rolC*. This suggests that *rolC *was the gene with the highest levels of expression and expression levels of *GAPDH *was the lowest, in this study.

**Figure 7:**
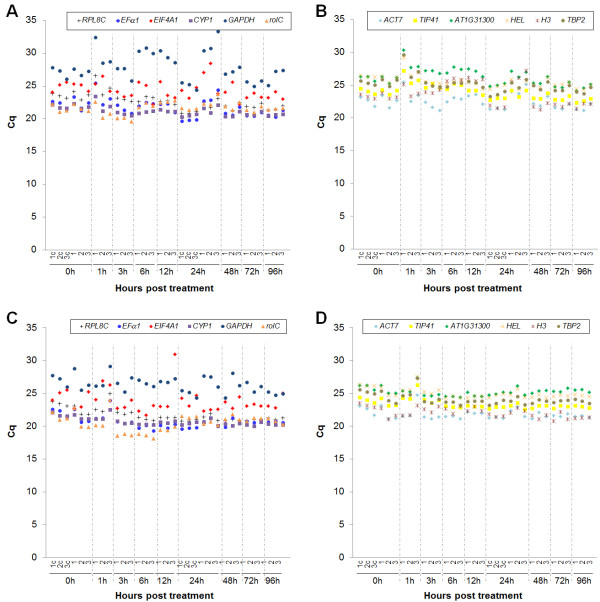
**Cq distribution of each reference gene among the 60 samples**. Cq distribution was performed for (**A**, **B**) NaOAc treatment and (**C**, **D**) MeJA treatment. Numbers 1 to 3 represent biological replicates. Numbers followed by a "c" represent control samples.

Variation of Cq values among the 33 samples for the 12 candidate reference genes is shown in Figure 7. Cq values among the samples varied less for the MeJA than the NaOAc treatment (Figure 6). It suggests that NaOAc treatment produced more changes in the expression levels of the analyzed candidate reference genes than MeJA, indicating that reference genes are regulated due to the treatment. Gene expression (Cq values) of the 12 genes varied across the time course experiments and also variation was observed against biological replicates (Figure 7). Variation between biological samples indicated that each individual responds in a unique manner under specific conditions. For reference gene selection the goal is to find a set of genes which have the lowest variation of gene expression between biological samples and under different conditions.

### Establishing gene expression stability of candidate reference genes

We used two algorithms, geNorm [35] and NormFinder [36] for selecting the best reference gene for our peanut hairy root cultures upon NaOAc and MeJA treatments. We used these algorithms to analyze our data under three modes: samples only treated with NaOAc; samples only treated with MeJA; and the complete set of samples/treatments that included treatment with NaOAc, MeJA and control conditions.

#### a) geNorm analysis

**Figure 8:**
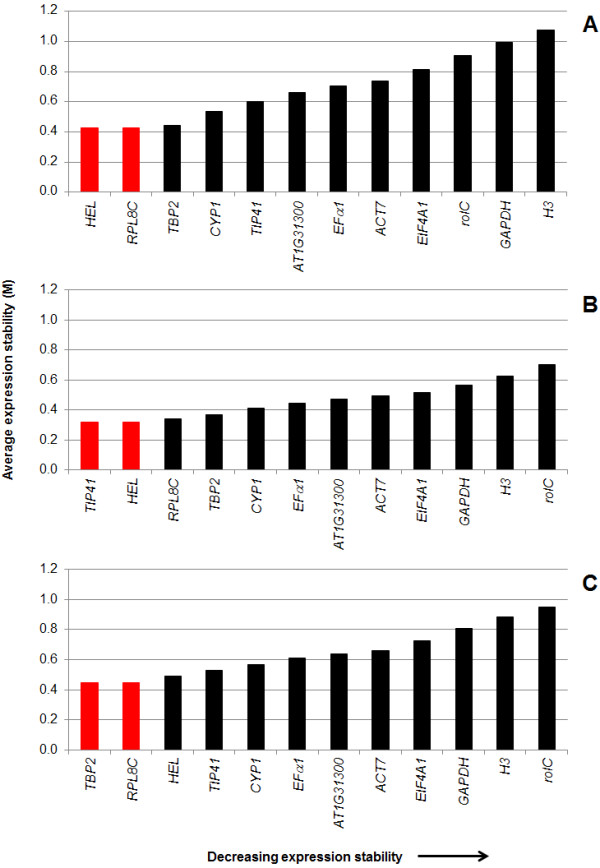
**Average expression stability values (M) of reference genes using geNorm**. M values for NaOAc treatment (**A**), MeJA treatment (**B**) and all treatments (controls and elicitors) containing the 60 biological samples are shown (**C**). Red bars indicated the best pair of reference genes calculated by the software.

GeNorm algorithm ranks the analyzed reference genes based on their expression stability (M), selecting an optimal pair of reference genes out of a larger group of candidate genes. The algorithm calculates and compares the M-value of the set of candidate genes, then eliminates the candidate gene with the highest M-value (more variation in gene expression) and repeats this procedure until two genes are left. This last pair of genes is recommended as the optimal pair of reference genes. This algorithm assumes that candidate genes are not co-regulated. As shown on Figure 8, the order of the ranking of gene stability was different between NaOAc and MeJA. The ranking of NaOAc for stable expression levels gave to *HEL *>*PRL8C *>*TBP2 *>*CYP1 *>*TIP41 *as the best five reference genes (Figure 8A). *HEL *and *RPL8C *were the most stable pair of reference genes under NaOAc treatment over the time course with M-value of 0.43. On the other hand, the gene with the least stable expression was *H3 *(M = 1.07). In the case of MeJA treatment time course, the ranking of reference genes was *TIP41 *>*HEL *>*PRL8C *>*TBP2 *>*CYP1 > EFα1 *as the best five reference genes (Figure 8B). In this case, the best pair of reference genes was *TIP41 *and *HEL *with M-value of 0.32; while the least stable expression was associated with *rolC *(M = 0.70) gene. Comparison of M-values for best pair of reference gene under each elicitation treatment showed that under MeJA more expression stability (less M value) was obtained even for the same gene (*HEL*). This was also observed for the 11 remaining reference genes; which correlated the difference in variation between treatments (Figure 6). Potato exposed to different stress conditions also showed that expression stability (M) values were dependent of the stress condition [27].

When all the treatments (controls and elicitors) were analyzed their combined expression stability ranking showed that the best five reference genes (*TBP2 *>*PRL8C *>*HEL *>*TIP41 *>*CYP1*) (Figure 8C) were the same genes observed under NaOAc and MeJA treatment. This order of gene ranking was more similar to the MeJA treatment. The best pair of reference genes was *TBP2 *and *RPL8C *(Figure 8C) with M-value of 0.45, and the least stable reference gene was *rolC *(M = 0.95). The M-value of 0.45 was the highest observed for the best pair of reference genes, compared to NaOAc (0.43) and MeJA (0.32). GeNorm considers high reference target stability for M-values ≤ 0.5 (for homogeneous samples, e.g. untreated cell culture). In all samples/treatments analysis the first three genes (*TBP2, PRL8C *and *HEL*) with low M-values fell into this category.

#### b) NormFinder analysis

**Figure 9:**
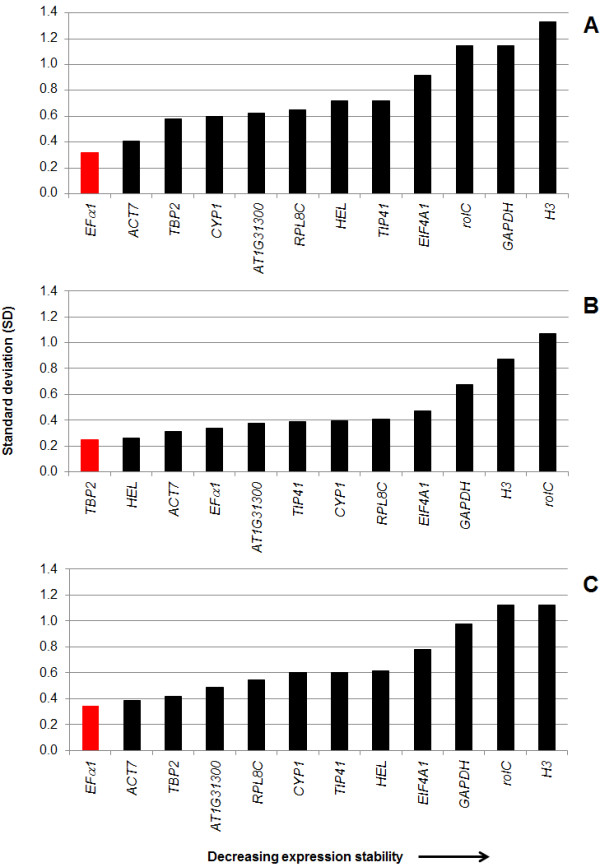
**Standard deviation (SD) of reference genes using NormFinder**. SD values for NaOAc treatment (**A**), MeJA treatment (**B**) and all treatments (controls and elicitors) containing the 60 biological samples are shown (**C**). Red bars indicated the best reference gene calculated by the software.

NormFinder also calculates an expression stability value (standard deviation) for the candidate reference genes. This method is similar to geNorm in that a low value of SD represents a more stable expression of the gene. This algorithm attempts to identify the optimum reference gene in a group of candidate genes. In contrast to geNorm, NormFinder produces a ranking of the genes taking information from their estimated intra- and intergroup variations. Analysis using this algorithm was performed for NaOAc (Figure 9A), MeJA (Figure 9B) and for all treatments (controls and elicitors) (Figure 9C). The ranking of reference genes for NaOAc gave as the best five genes to *EFα1 > ACT7 *>*TBP2 *>*CYP1 *>*AT1G31300 *(Figure 9A). The best reference gene was *EFα1 *with a SD-value of 0.32 and the gene with the least stable expression was *H3 *(SD = 1.33). For MeJA treatment the best five reference genes were *TBP2 *>*HEL *>*ACT7 *>*EFα1 *>*AT1G31300 *(Figure 9B). In this case, *TBP2 *was the best reference gene with a SD-value of 0.25 and the gene with the least stable expression was *rolC *(SD = 1.07). The ranking for reference genes between NaOAc and MeJA was different. Similar to the finding when geNorm was applied, SD values for MeJA were lower than NaOAc suggesting that less variability was observed under MeJA treatment. When the analysis was performed for all the treatments (controls and elicitors), the best five reference genes were *EFα1 *>*ACT7 *>*TBP2 *>*AT1G31300 *>*PRL8C *(Figure 9C). The best reference gene was *EFα1 *with a SD-value of 0.35 and the genes with the least stable expression were *H3 *and *rolC *(SD = 1.12). No optimal SD-values for NormFinder have been specified. This order of the ranking of reference genes (all treatments) was similar to the NaOAc treatment.

#### c) Identifying most optimal reference genes based on cumulative geNorm and NormFinder analyses

The difference on selection of reference gene between these two algorithms is that when geNorm eliminates the gene with the largest variation, after selection procedure, also it does not consider the Cq values from this gene for further analysis. The implications of this are that different sizes of groups are analyzed across the procedure thus recalculating the standard deviation at each step. This results in the standard deviation being different after each elimination process of the gene with the largest variation. In contrast, NormFinder is based on ANOVA (analysis of variance); which considers all the Cq values, from the genes analyzed, through the entire process of selection of reference gene.

The best reference genes considering all treatments (controls and elicitors) were *TBP2 *and *RPL8C*, and *EFα1 *for geNorm (Figure 8C) and NormFinder (Figure 9C), respectively. *TBP2 *and *RPL8C *were placed in the middle top on NormFinder ranking (third and fifth, respectively). In the case of *EFα1*, it placed sixth on the geNorm ranking. It is interesting to note that *TBP2, RPL8C *and *EFα1 *placed in the top or middle of the ranking in previous studies in which different plant tissue (leaf and root) [15], developmental stages [28-30,33] or stress conditions (biotic and abiotic) [27] were employed. *EFα1 *has been shown to be one of the most stable reference genes in qPCR [27-29,33] and our study confirmed that. Although *RPL8C *is not a conventional reference gene, it has been used as reference gene in humans [12]. This gene was placed in the middle of the ranking of reference genes in prior studies [27,30]. Interestingly, when stress conditions (late blight exposure, cold stress and salt stress) were evaluated in potato for validation of reference genes, *RPL8C *was ranked on the top middle [27]. This suggests that *RPL8C *may prove to be a good target for other plant stressors on other plant species. On the other hand, *TBP2 *was used in a previous study, in which it placed the fourth position in the ranking of reference genes [30].

*GAPDH *is commonly used as reference gene in animals [67,68] and plants [69]. In peanut hairy roots, *GAPDH *was shown to be one of the least stable expression reference genes tested (Figures 8 and 9) and this was independent of the algorithm used. This has been shown to be the case as well for other plant RT-qPCR studies [13,15,30,33]. Together these results reinforce the concept that some conventional reference genes are not good choices for normalization of gene expression and the importance of always confirming your reference genes for the specific system you are studying. Of the 21 genes screened, *H3 *and *rolC *showed greater variation in expression levels using either algorithm.

### Optimal number of reference genes for normalization

**Figure 10:**
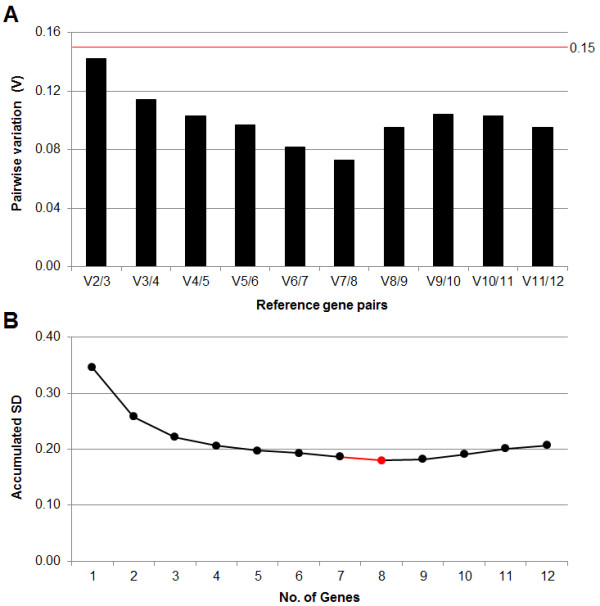
**Optimal number of reference genes for normalization**. Calculations were performed using pairwise variation, calculated on geNorm (**A**) and accumulated SD, calculated on NormFinder (**B**). For **A**: V2/3, pairwise variation between the two most stable genes (*TBP2 *and *RPL8C*) + 3^rd ^most stable gene (*HEL*); V3/4, addition of the 4^th ^most stable gene (*TIP41*); V4/5, addition of the 5^th ^most stable gene (*CYP1*); V5/6, addition of the 6^th ^most stable gene (*EFα1*); V6/7, addition of the 7^th ^most stable gene (*AT1G31300*); V7/8, addition of the 8^th ^most stable gene (*ACT7*); V8/9, addition of the 9^th ^most stable gene (*EIF4A1*); V9/10, addition of the 10^th ^most stable gene (*GAPDH*); V10/11, addition of the 11^th ^most stable gene (*H3*); V11/12, addition of the 12^th ^most stable gene (*rolC*). For **B**: 1, *EFα1*; 2, 1 + *ACT7*; 3, 2 + *TBP2*; 4, 3 + *AT1G31300*; 5, 4 + *PRL8C*; 6, 5 + *CYP1*; 7, 6 + *TIP41*; 8, 7 + *HEL*; 9, 8 + *EIF4A1 *; 10, 9 + *GAPDH*; 11, 10 + *rolC*; 12, 11 + *H3*.

Normalization procedure for quantification of gene expression is crucial for accuracy of expression levels of target genes. The most common method for normalizing mRNA data is the use of reference genes. However, the choice of reference gene and how many are needed for normalization is a concern. In order to determine the optimal number of reference genes for our hairy root system, two algorithms were analyzed. The first one was calculated by geNorm (pairwise variation: V) (Figure 10A) and the second one was calculated by NormFinder (accumulated SD) (Figure 10B).

Pairwise variation (V) is calculated based on normalization factor values (NF_n _and NF_n+1_) after the inclusion of a least stable reference gene and indicates if the extra reference gene adds to the stability of the normalization factor. A threshold V-value of 0.15 is recommended by qbasePLUS software as optimal to determine the minimum number of reference genes. In the present study, the analyses used suggest a minimum of two reference genes (*TBP2 *and *RPL8C*) were needed to be below the V-value of 0.15. The lowest V-value (0.073) was obtained when the addition of the 8^th ^most stable gene (*ACT7*) was done (Figure 10A). Addition of more genes increased the V-values, indicating that those least stable reference genes will negatively impact the normalization process. Our results have shown that only two reference genes are needed to be below the threshold value. Previous studies in which determination of optimal number of reference genes was done showed that even using four [15] or seven [31] of their best reference genes, the V-values were not below the threshold V-value.

Accumulated SD is an indicator of the optimal number of reference genes. The optimal number of reference genes described by NormFinder was 8 (Figure 10B), taking into account those genes up to the 8^th ^most stable reference gene (*HEL*) (Figure 9C). Similar to pairwise variation, addition of the least stable reference genes will increase the variability, which must be avoided. Often, using 8 reference genes for normalization procedures is not experimentally practical (use of more reagents and time consuming). In this case consideration must be taken to determine how much accumulated (acc.) SD decreases when an additional reference gene is added to the normalization procedure. For example, in Figure 10B when one reference gene is used the acc. SD was 0.345. This value dropped to 0.258 when the second reference gene was added, a difference of 0.087 of acc. SD. When the third reference gene is considered, the acc. SD decreased to 0.221 (a difference of 0.037). However, when the fourth reference gene is added acc. SD only decreased in 0.015. The "rate of change" in SD between each gene when no longer increasing or maintain is generally a good indicator that the number of reference genes required has been achieved. Therefore, the first three most stable reference genes could be used for normalization instead of 8 genes, because the most acc. SD is eliminated with the first 3 reference genes.

It is also important to consider the other sources of variation in the qPCR process. One possible contributor is the real-time PCR instrument. In the case of this block cycler, the well-to-well variation (SD) has been calculated to be ± 0.20 by the manufacturer (CFX384™ Real-time detection system, Bio-Rad). Considering that a variation (SD) of 0.20 is present when the qPCR instrument is used, there is no reason in adding more than 4 reference genes (in this study, Figure 10B) for normalization if there is a "default" variation during the qPCR process due to the instrument.

## Conclusion

This study provides the first validation of reference genes for RT-qPCR in hairy root cultures. Selection of an appropriate RNA extraction method to yield adequate amounts of RNA for RT-PCR was critical. Twenty-one candidate reference genes were measured in peanut hairy root cultures treated with two elicitors (NaOAc and MeJA). Due to poor PCR efficiencies, nine of the 21 genes were discarded. Analysis of the relative expression stability of reference genes using geNorm and NormFinder resulted in different reference genes being designated as lead targets. However, overall the *TBP2 *was the most stable across both elicitation NaOAc and MeJA treatments, followed by *RPL8C. TBP2 *is a non-traditional reference gene and we recommend testing its utility for not only normalization of gene expression measurements in peanut hairy roots under stress conditions as well as possibly other plant stress conditions. Interestingly, *TBP2 *is a TATA binding protein required for basal transcription in the cell. It functions as a transcription factor that binds to DNA sequence known as TATA box during the transcription process, thus having a steady state level of expression in the cell under different conditions. In addition, data analysis showed that the evaluated genes had more variation after NaOAc than MeJA treatment. The transgene (*rolC*) was also evaluated as reference gene and found to be one of the genes with low expression stability. The minimum number of reference genes for normalization was calculated to be two genes (*TBP2 *and *RPL8C*) using geNorm and three genes (*EFα1, ACT7 *and *TBP2*) using NormFinder. It is possible that the other genes that were eliminated from the analysis based on PCR efficiency may be good candidates as reference genes if qPCR primers were redesigned in other regions of the target gene sequence. Such assessment will be facilitated with the full sequence of the peanut genes analyzed which currently is not available. In future studies using peanut hairy root elicited with NaOAc or MeJA, *TBP2 *and *RPL8C *are recommended to be used as reference genes.

## Methods

### Plant material and elicitor treatments

All experiments were conducted with hairy root cultures of peanut (*Arachis hypogaea*) cv. Hull line 3 [48]. Briefly, the cultures were established by direct inoculation of stem explants with *Agrobacterium rhizogenes *strain ATCC 15834. Hairy roots developed at the inoculation site after 2 weeks. Line 3 used in this study derived from one initial hairy root that developed at the *Agrobacterium *inoculation site and was clonally propagated using root tips. The hairy root line was maintained by subculturing 10 root tips into 250 mL flasks containing 50 mL of a modified MS medium (MSV) as previously described [48]. At day 9 of culture (mid-exponential growth stage), the spent medium was replaced with fresh MSV medium containing as elicitors 10.2 mM NaOAc [47] or 100 μM MeJA. As controls, the medium of 9-day cultures was replaced with MSV medium without elicitor. The tissue was collected at 0, 1, 3, 6, 12, 24, 48, 72 and 96 h after elicitor treatment (NaOAc and MeJA), then frozen at -80°C and lyophilized as previously described [48].

### RNA extraction

Total RNA was extracted from lyophilized tissue with TRIzol^® ^(ratio of 20 mg DW tissue to 1 mL solvent) (Invitrogen) according to manufacturer's procedure. After extraction, the RNA was dissolved in 50 μL of DNase/RNase-free distilled water (ultraPURE™, Gibco). Genomic DNA contamination was eliminated by treating the RNA with TURBO DNA-*free*™ (Applied Biosystems).

RNA concentration was determined using Quant-iT™ RiboGreen^® ^RNA kit (Invitrogen) using the following modified method for a 96-well microplate format. A standard curve was generated using seven serial dilutions (1:2). The concentrations ranged from 1 μg/mL to 15.63 ng/mL. One hundred μL of standard or sample and 100 μL of diluted Quant-iT™ RiboGreen^® ^reagent were used in 200 μL of assay volume. Standards were run in triplicate on each plate. Samples were analyzed at two concentrations (dilution 1:5). Detection was done by fluorescence (excitation at 485 nm) and emission at 520 nm) using a POLARstar OPTIMA microplate reader (BMG Labtech).

Purity of the total RNA extracted was estimated from the ratio of absorbance readings at 260 and 280 nm using a NanoDrop™ 800 spectrophotometer (Thermo Scientific).

### Comparison of RNA extraction methods

Total RNA extracted with Maxwell^® ^16 Total RNA Purification kit (Promega) was done according to the manufacturer's procedure described for plant tissue samples with the following modifications for lyophilized tissue. Samples (10, 20 or 40 mg DW) were treated with 500 μL of Lysis buffer. After lysis step, the lysate volumes obtained were 500, 400 and 300 μL for 10, 20 and 40 mg, respectively. The amount of Blue RNA Dilution Buffer added to the sample lysate was according to manufacturer's procedure. The same amount of Clearing Agent (125 μL) was added independent of the amount of DW used. Alternatively, BME was added into the Lysis Buffer in the Maxwell^® ^procedure. Then, samples were loaded into Maxwell^® ^16 Total RNA Purification cartridge (Promega) and processed using the Maxwell^® ^16 MDx instrument (Promega) following the default protocol for RNA extraction. Contaminating genomic DNA was eliminated through the Maxwell^® ^16 Total RNA Purification kit.

Total RNA quantification was done using Quant-iT™ RiboGreen^® ^RNA kit, as previously described. Final volumes of pure RNA were 50 and 220 μL for TRIzol^® ^and Maxwell^®^, respectively.

### RNA integrity

Nine-day peanut hairy root cultures were elicited with NaOAc 10.2 mM [47]. Control cultures did not include NaOAc. After 3 hours of treatment the roots were frozen with liquid nitrogen and either stored at -80°C or lyophilized as previously described [48]. Three biological replicates per treatment/storage were considered. Total RNA extraction was done with TRIzol^® ^as previously described, except that RNA was treated with RQ1 RNase-Free DNase (Promega). As starting material, 40 mg and 100 mg were used of lyophilized and frozen tissue, respectively. Two technical replicates of RNA extraction were done. RNA was quantified by absorbance at 260 nm using a spectrophotometer (ND-1000, Nanodrop^®^). RNA was run on 1.2% agarose gel electrophoresis containing 2.2 M formaldehyde as previously described [70]. Technical replicates of RNA extractions were pooled and an average of their concentrations was considered for further analysis. Two μg of RNA per sample were loaded on agarose gel. SYBR^® ^Gold NucleicAcid Gel Stain (Invitrogen) was used to visualize RNA following the manufacturer's procedure.

### Selection of reference genes

Twenty-one candidate reference genes were selected as shown in Table 1. These genes were involved in different functional classes in the cell. This group of genes comprised five commonly used reference genes: *TUA3 *(α-tubulin), *ACT7 *(actin 7), *EFα1 *(elongation factor α1), *GAPDH *(glyceraldehyde-3-phosphate dehydrogenase) and *UBQ11 *(ubiquitin 11). Less common reference genes such as *H3 *(histone H3), *TBP2 *(TATA binding protein 2), *RPB1 *(RNA polymerase II large subunit), *RPL8C *(60S ribosomal protein L8), *EIF4A1 *(eukaryotic translation initiation factor 4A1), *CYP1 *(cyclophilin) and *APT1 *(adenine phosphoribosyltransferase) were also included.

The set of candidate reference genes also included less conventional reference genes which expression levels showed to be more stable in an analysis of microarray data-sets from *Arabidopsis *[13]. These genes included: *TIP41 *(Tip41-like family protein), *SAND *(SAND family protein), *AP47 *(clathrin-associated protein), *AT4G33380 *(expressed sequence), *PP2AA3 *(protein phosphatase 2A subunit A3), *HEL *(helicase domain-containing protein) and *AT1G31300 *(expressed sequence). Most of these genes also showed stable expression in other plant species [29,30].

To identify peanut homologous sequence to the candidate reference genes, TBLASTN analysis from the National Center for Biotechnology Information (NCBI) was used. A query for *Arachis hypogaea *(taxid3818 [ORGN]) nucleotide sequences [nucleotide collection (nr/nt)] with *Arabidopsis *protein sequences was performed. In the case of *H3*, first a homologous gene in *Arabidopsis *was obtained using *Ziziphus jujuba *protein sequence [GenBank: ACG70966] [32]. Although a similar approach was followed for the *RPB1 *sequence, a human (*Homo sapiens*) protein sequence [GenBank: NP_000928] was used. Due to lack of a peanut sequence that matched the *Arabidopsis *homologous locus AT4G38710 [26] (additional file 1); another homologous gene to *Arabidopsis *was obtained using *Oryza sativa *protein sequence for *EIF4A1 *[GenBank: BAG93556.1] [28].

Transgenes that harbor the T-DNA from *Agrobacterium rhizogenes *were also included in the set of candidate reference genes: *rolC *(root loci C gene) and *aux1 *(tryptophan oxygenase gene). Sequences for *rolC *and *aux1 *genes were obtained from the GenBank (NCBI) (Table 1).

### Primer design

Protein coding gene models for all the candidate reference genes, except *rolC *and *aux1*, were obtained for *Arabidopsis thaliana *from the TAIR (The *Arabidopsis *Information Resource) web site http://www.arabidopsis.org. In each case the latest version or the most conserved gene model was used. *Arabidopsis *nucleotide sequences for mRNA, genomic DNA (gDNA) and amino acid sequence were downloaded from the GenBank (NCBI) (Table 1). An alignment between the gDNA and mRNA (for the same gene) for *Arabidopsis *sequences was done using AlignX (Vector NTI^®^) with the purpose of localizing intron regions. Intron regions were double-checked manually to confirm that they follow the GT-AG, CC-AG or AT-AC intron rules [71]. Based on the protein coding gene model for *Arabidopsis*, two genes did not present introns in their sequences: *H3 *and *CYP1*. The peanut sequence obtained for each gene from TBLASTN was aligned against the previous alignment between mRNA - gDNA (*Arabidopsis *sequence) to determine exon position in the peanut sequences.

SYBR^® ^green primers for qPCR were designed using AlleleID^® ^7 software (Premier Biosoft). Primers flanking intron regions were designed, when gene sequence contained introns. Secondary DNA structures have been shown to affect PCR efficiency [72], therefore peanut sequences were analyzed for secondary structure to avoid these regions for primers design. For sequences longer than 1200 bp the "mfold" website http://mfold.rna.albany.edu/?q=mfold/DNA-Folding-Form was used. The structures were downloaded in "rnaml" format and loaded into AlleleID^® ^7. Gene sequences were blasted against nr database (GenBank, NCBI) to determine cross homology with other sequences (target specificity). After these analyses were done for each of the 21 candidate reference gene, primers were designed for each gene.

### RT-qPCR

Two-step RT-qPCR was performed using SYBR Green detection chemistry. cDNA was synthesized from 1 μg of total RNA and oligo(dT) primers, using iScript™ Select cDNA Synthesis kit (Bio-Rad), following the manufacture's procedure. Volume of RNA treated with TURBO DNA-*free*™ was less than 25% of the final RT-PCR volume; because no more than 40% of the final volume can be used, otherwise some inhibition of the qPCR reactions might occur according to manufacturer's recommendation. Quantitative real-time PCR reactions were prepared in a total volume of 10 μL containing: 4 μL of template (10 ng) and 6 μL of master mix [0.4 μL of each primer (0.8 μM, final concentration), 5 μL of iQ™ SYBR^® ^Green Supermix (Bio-Rad) (1X, final concentration) and 0.2 μL of ultraPURE™ water]. Primers were synthesized by Invitrogen. Pipetting was performed using the epMotion 5075 (Eppendorf) on Hard-Shell^® ^Thin-Wall 384-Well Skirted PCR plates (Bio-Rad) sealed with Microseal^® ^'B' adhesive seal (Bio-Rad). Technical samples were run in triplicate at the RT level. Non-template controls (NTC) were run in three technical replicates. All qPCRs were performed using the CFX384™ Real-time PCR Detection System (Bio-Rad). The following amplification program was used: denaturation at 95°C for 3 min, 40 cycles of amplification (95°C for 10 s, 60°C for 30 s) and a melting curve program (from 65°C to 95°C, with an increment of 0.5°C for 5 s). Data were collected using the CFX Manager 2.0 (Bio-Rad). Minus reverse transcription (-RT) control, which assesses the presence of genomic contamination in the sample, was not used in this study. Genomic DNA was eliminated with DNase treatment. To confirm that no contaminant genomic DNA was present after DNase treatment, analysis of *ACT7 *was done. Primers for *ACT7 *were designed flanking an intron of 87 bp. As shown in Figure 5 the size of the amplicon corresponded to the predicted cDNA size (Table 1), indicating no presence of contaminating DNA.

For PCR efficiencies a pool of 6 samples from the time course experiment were used (3 elicited and 3 control samples). RNA extraction and their treatment with DNase were made separately for each sample as already detailed. RNA was quantified for each sample with Quant-iT™ RiboGreen^® ^RNA kit, and RNA samples were pooled, quantified and used for cDNA synthesis as previously described. The cDNA used as PCR template was in a range of 40, 8, 1.6, 0.32, 0.064 and 0.0128 ng (1:5 dilution series). Quantitative PCR reactions and conditions were as described above, except that 45 cycles were run instead of 40 cycles. Quantitative PCR amplifications were run using three technical replicates. NTC was run in three technical replicates per gene. Efficiency (E) for each gene was determined with the slope of a linear regression model [73] using the Cq values and the following equation was used:

$$\text{E}=10^{\left[-1∕\text{slope}\right]}-1$$

The sample used for assessing PCR efficiency (1.6 ng) with the *RPL8C *gene was employed as an inter-plate calibrator when the 12 sets of primers were run. The Cq value of the inter-plate calibrator was ~ 25 and it was run in three technical replicates per plate. This sample was selected from data generated for PCR efficiency.

PCR product sizes were checked on 3% agarose gel and ethidium bromide staining. Melting curves were analyzed for each gene using CFX Manager Version 2.0.; qPCR efficiency between 90 and 110%, r^2 ^≥ 0.95, a single peak in the melting curve were requirements for considering a gene as a good candidate.

### Analysis of gene expression stability

To analyze gene expression stability and rank, geNorm [35] and NormFinder [36] algorithms were used. They were included on GenEx (multid) software. GeNorm V (pairwise variation) was performed using qbasePLUS Version 1.5.

### Statistical analysis

Cq value comparison of the genes between treatments was calculated with ANOVA using a Bonferroni correction and a significance cut-off of 0.01. Comparison of RNA yields was calculated with ANOVA using the Tukey's test with a significance cut-off of 0.05. These analyses were performed using GraphPad Prisma^® ^software.

## Abbreviations

BME: β-mercaptoethanol; cDNA: complementary DNA; Cq: quantification cycle; DW: dry weight; EST: expressed sequence tag; MeJA: methyl jasmonate; MIQE: minimum information for publication of quantitative real-time PCR experiments; mRNA: messenger RNA; NaOAc: sodium acetate; NTC: no template control; PCR: polymerase chain reaction; qPCR: quantitative real-time PCR; RB: right border; RT-PCR: reverse transcription PCR; RT-qPCR: reverse transcription quantitative real-time PCR; SD: standard deviation; SRA: sequence real archive; T-DNA: transfer DNA; T_R_-DNA: right T-DNA

## Competing interests

The authors declare that they have no competing interests.

## Authors' contributions

JC performed data analysis, carried out the statistical analysis and drafted the manuscript; JC and CNO carried out the entire experimental procedure; JC and FMB conceived of the study, designed and evaluated the experiments. GM supervised experimental design and evaluated the experiments. All authors read, revised and approved the final manuscript.

## Supplementary Material

Additional file 1 **Summary of reference genes in literature used in plants and humans^1^**. Select literature used in identifying potential validation of reference genes candidates are listed by first author and year of the publication. Gene targets used for each study are indicated by accession number (GenBank or TAIR database). One reference^1 ^provides reference genes used for humans. The gene(s) identified as best reference gene(s) in each of the cited studies is showed in red. In green: Genes selected from Transcript arrays in *Arabidopsis *[13]. In yellow: Most frequently reference genes used. Click here for file

Additional file 2 **RNA chemical contaminants**. Indicator of chemical contaminants in RNA was measured using the ratio of: absorbance at 260 nm/absorbance at 230 nm. X-axis shows the intervals of A_260_/A_230 _for the 60 biological samples from the time course experiments. Click here for file

Additional file 3 **Primer specificity of reference genes for *APT1, UBQ11 *and *AP47***. Melting curves generated for 40 (red), 8 (green), 1.6 (light blue), 0.32 (yellow) and 0.064 (black) ng of cDNA. Click here for file

Additional file 4 **Primer specificity of reference genes for *ACT7, TIP41 *and *H3***. Melting curves generated for 40 (red), 8 (green), 1.6 (light blue), 0.32 (yellow) and 0.064 (black) ng of cDNA. Click here for file

Additional file 5 **Primer specificity of reference genes for *TBP2, EFa1 *and *RPL8C***. Melting curves generated for 40 (red), 8 (green), 1.6 (light blue), 0.32 (yellow) and 0.064 (black) ng of cDNA. Click here for file

Additional file 6 **Primer specificity of reference genes for *EIF4A1, CYP1 *and *rolC***. Melting curves generated for 40 (red), 8 (green), 1.6 (light blue), 0.32 (yellow) and 0.064 (black) ng of cDNA. Click here for file

Additional file 7 **Primer specificity of reference genes for *AT1G31300, HEL *and *GAPDH***. Melting curves generated for 40 (red), 8 (green), 1.6 (light blue), 0.32 (yellow) and 0.064 (black) ng of cDNA. Click here for file

Additional file 8 **Primer specificity of reference genes for *TUA3, SAND *and *RPB1***. Melting curves generated for 40 (red), 8 (green), 1.6 (light blue), 0.32 (yellow) and 0.064 (black) ng of cDNA. Click here for file

Additional file 9 **Primer specificity of reference genes for *AT4G33380, PP2AA3 *and *aux1***. Melting curves generated for 40 (red), 8 (green), 1.6 (light blue), 0.32 (yellow) and 0.064 (black) ng of cDNA. Click here for file
