# Supplementary figures and images for: Selection of reference genes for qPCR in hairy root cultures of peanut
Source: BMC Res Notes. 2011 Oct 10;4:392. doi: 10.1186/1756-0500-4-392 (PMC3199266; doi:10.1186/1756-0500-4-392)

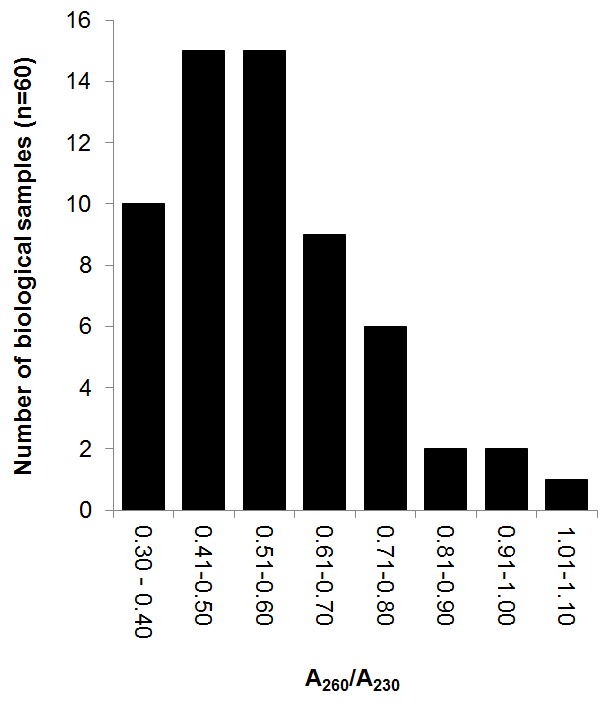

Supplement: Additional file 2 — RNA chemical contaminants. Indicator of chemical contaminants in RNA was measured using the ratio of: absorbance at 260 nm/absorbance at 230 nm. X-axis shows the intervals of A260/A230 for the 60 biological samples from the time course experiments. [file 1756-0500-4-392-S2.PNG]

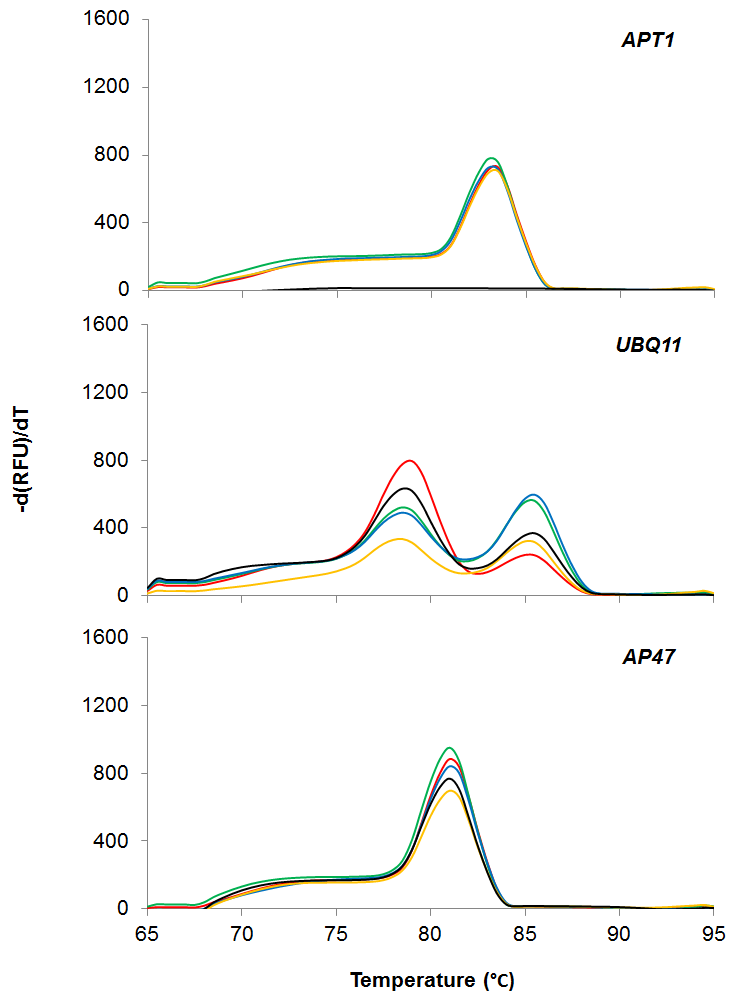

Supplement: Additional file 3 — Primer specificity of reference genes for APT1, UBQ11 and AP47. Melting curves generated for 40 (red), 8 (green), 1.6 (light blue), 0.32 (yellow) and 0.064 (black) ng of cDNA. [file 1756-0500-4-392-S3.PNG]

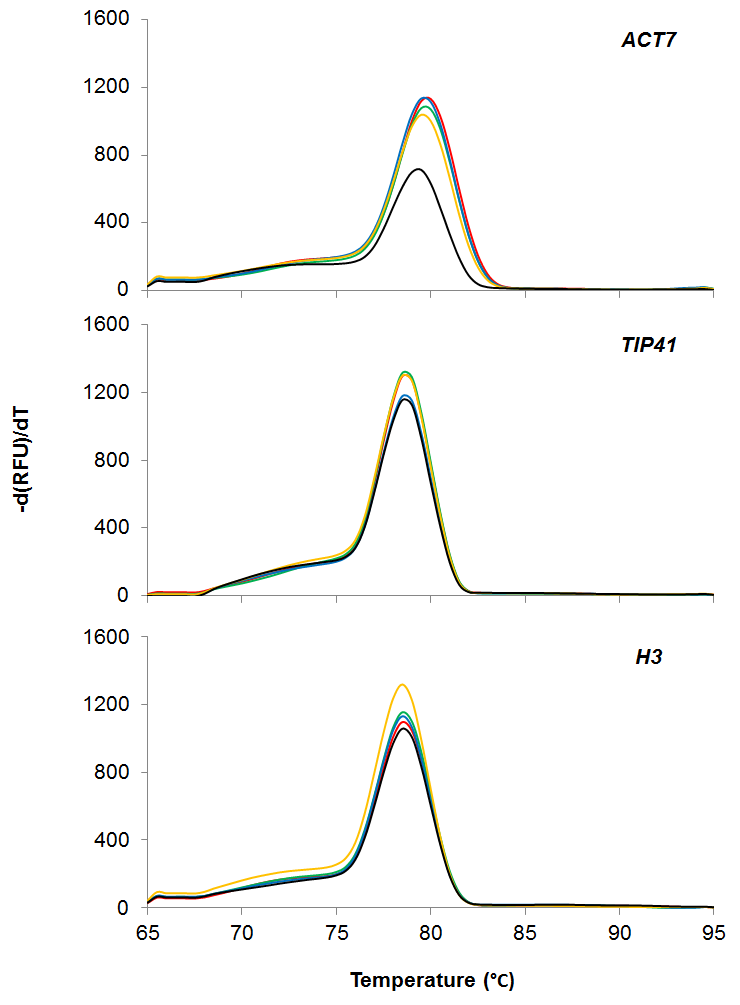

Supplement: Additional file 4 — Primer specificity of reference genes for ACT7, TIP41 and H3. Melting curves generated for 40 (red), 8 (green), 1.6 (light blue), 0.32 (yellow) and 0.064 (black) ng of cDNA. [file 1756-0500-4-392-S4.PNG]

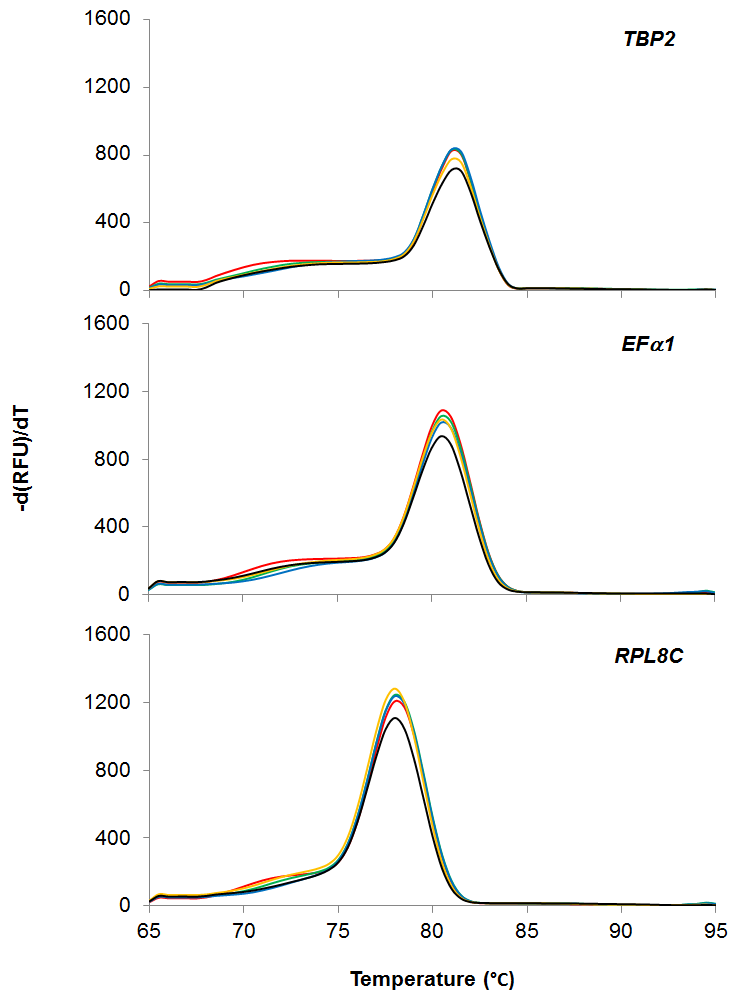

Supplement: Additional file 5 — Primer specificity of reference genes for TBP2, EFa1 and RPL8C. Melting curves generated for 40 (red), 8 (green), 1.6 (light blue), 0.32 (yellow) and 0.064 (black) ng of cDNA. [file 1756-0500-4-392-S5.PNG]

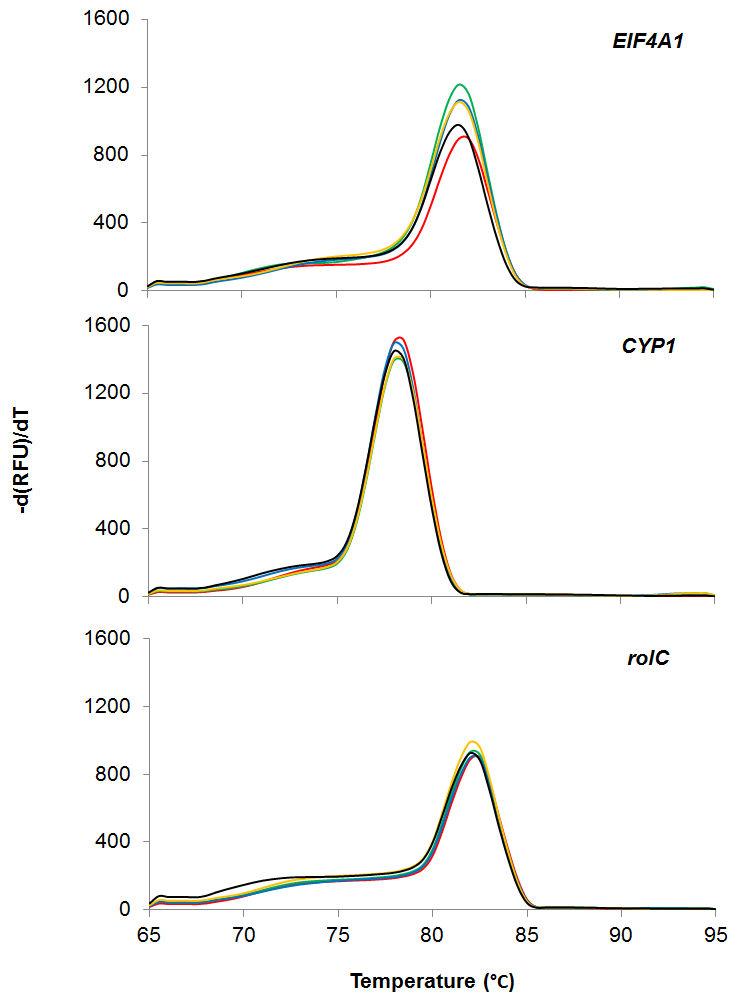

Supplement: Additional file 6 — Primer specificity of reference genes for EIF4A1, CYP1 and rolC. Melting curves generated for 40 (red), 8 (green), 1.6 (light blue), 0.32 (yellow) and 0.064 (black) ng of cDNA. [file 1756-0500-4-392-S6.PNG]

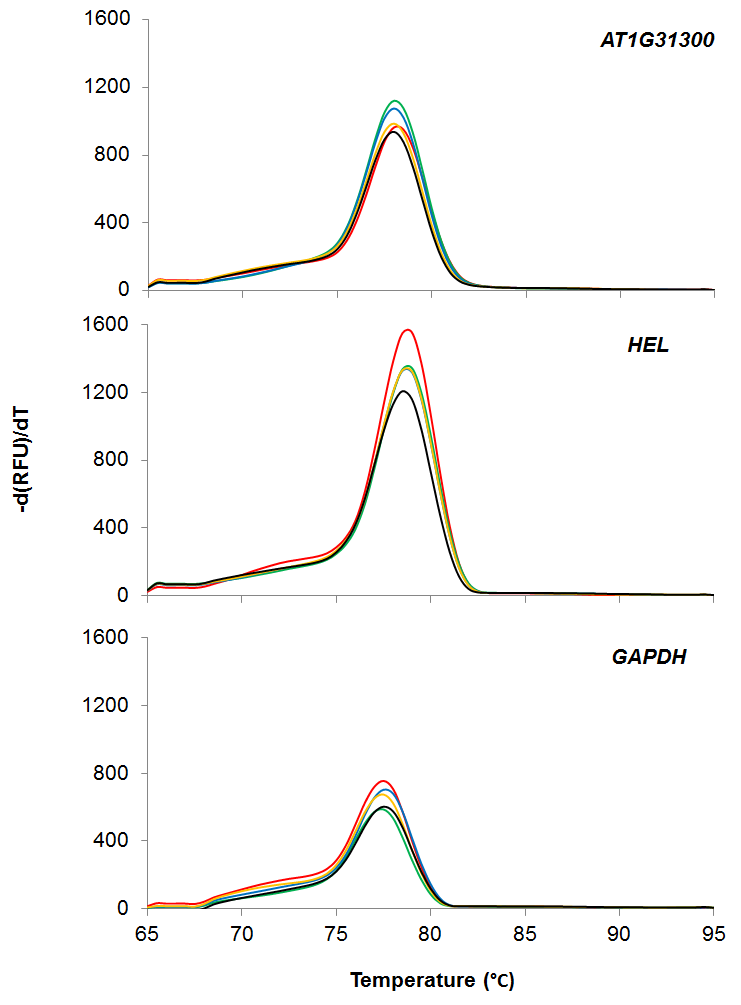

Supplement: Additional file 7 — Primer specificity of reference genes for AT1G31300, HEL and GAPDH. Melting curves generated for 40 (red), 8 (green), 1.6 (light blue), 0.32 (yellow) and 0.064 (black) ng of cDNA. [file 1756-0500-4-392-S7.PNG]

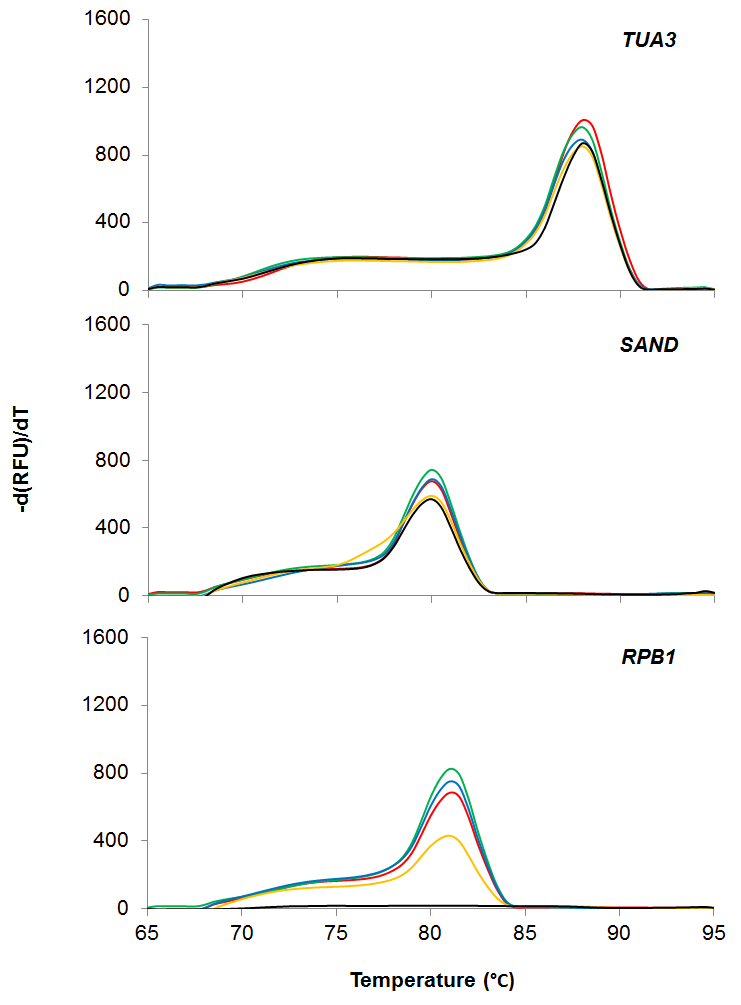

Supplement: Additional file 8 — Primer specificity of reference genes for TUA3, SAND and RPB1. Melting curves generated for 40 (red), 8 (green), 1.6 (light blue), 0.32 (yellow) and 0.064 (black) ng of cDNA. [file 1756-0500-4-392-S8.PNG]

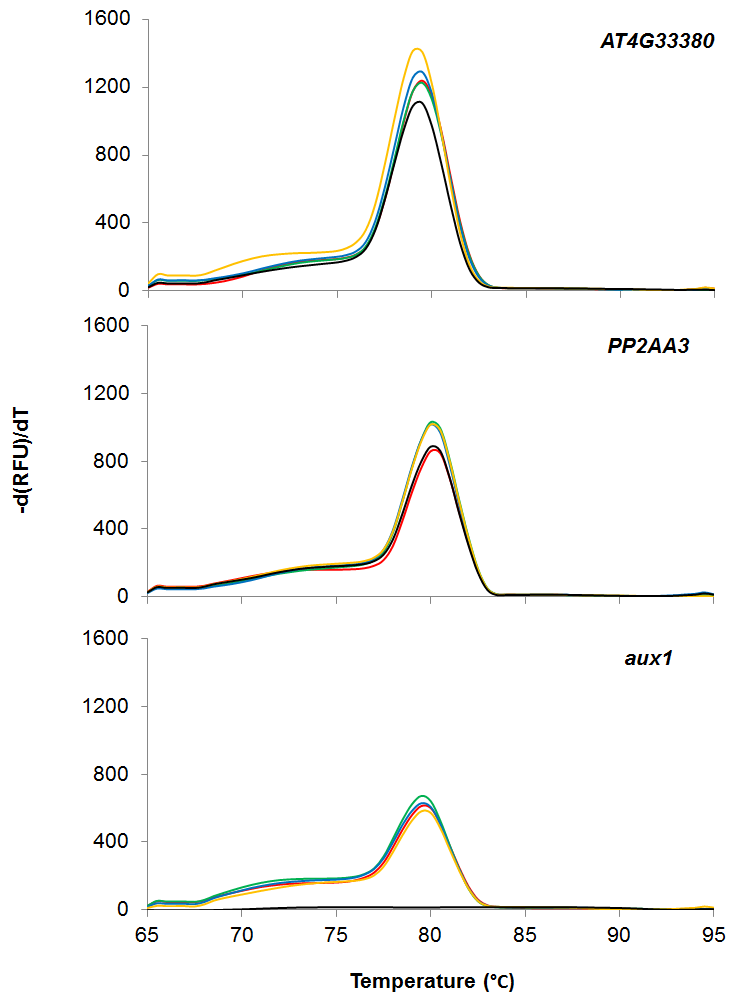

Supplement: Additional file 9 — Primer specificity of reference genes for AT4G33380, PP2AA3 and aux1. Melting curves generated for 40 (red), 8 (green), 1.6 (light blue), 0.32 (yellow) and 0.064 (black) ng of cDNA. [file 1756-0500-4-392-S9.PNG]
